# Polish Consumers’ Understanding of Different Front-of-Package Food Labels: A Randomized Experiment

**DOI:** 10.3390/foods11010134

**Published:** 2022-01-05

**Authors:** Valentina A. Andreeva, Manon Egnell, Katarzyna Stoś, Beata Przygoda, Zenobia Talati, Mathilde Touvier, Pilar Galan, Serge Hercberg, Simone Pettigrew, Chantal Julia

**Affiliations:** 1 Nutritional Epidemiology Research Group (EREN), Sorbonne Paris Nord University/INSERM U1153/INRAE U1125/CNAM, Epidemiology and Statistics Research Center, University of Paris (CRESS), 93017 Bobigny, France; m.egnell@eren.smbh.univ-paris13.fr (M.E.); m.touvier@eren.smbh.univ-paris13.fr (M.T.); p.galan@eren.smbh.univ-paris13.fr (P.G.); s.hercberg@eren.smbh.univ-paris13.fr (S.H.); c.julia@eren.smbh.univ-paris13.fr (C.J.); 2 National Institute of Public Health NIH-National Research Institute, 00-791 Warsaw, Poland; kstos@pzh.gov.pl (K.S.); bprzygoda@pzh.gov.pl (B.P.); 3 Western Australian Cancer Prevention Research Unit, School of Population Health, Curtin University, Bentley 6102, Australia; zenobia.talati@curtin.edu.au; 4 Department of Public Health, AP-HP Paris Seine-Saint-Denis Hospital System, 93017 Bobigny, France; 5 The George Institute for Global Health, University of New South Wales, Sydney 2042, Australia; spettigrew@georgeinstitute.org.au

**Keywords:** front-of-package label, food and beverage labeling, nutritional value, diet, randomized trial, Central-Eastern Europe, public health

## Abstract

Dietary practices are a key behavioral factor in chronic disease prevention; one strategy for improving such practices population-wise involves front-of-package labels (FoPL). This online randomized study, conducted in a quota-based sample of 1159 Polish adults (mean age = 40.9 ± 15.4 years), assessed the objective understanding of five FoPL: Health Star Rating, Multiple Traffic Lights, NutriScore, Reference Intakes (RI) and Warning Label. Objective understanding was evaluated by comparing results of two nutritional quality ranking tasks (without/with FoPL) using three food categories (breakfast cereals, cakes, pizza). Associations between FoPL exposure and objective understanding were assessed via multivariable ordinal logistic regression. Compared to RI and across food categories, significant improvement in objective understanding was seen for NutriScore (OR = 2.02; 95% CI: 1.41–2.91) and Warning Label (OR = 1.61; 95% CI: 1.12–2.32). In age-stratified analyses, significant improvement in objective understanding compared to RI emerged mainly among adults aged 18–30 years randomized to NutriScore (all food categories: OR = 3.88; 95% CI: 2.04–7.36; cakes: OR = 6.88; 95% CI: 3.05–15.51). Relative to RI, NutriScore was associated with some improvement in objective understanding of FoPL across and within food categories, especially among young adults. These findings contribute to the ongoing debate about an EU-wide FoPL model.

## 1. Introduction

The increase in life expectancy observed in Poland over the last few decades is a product of multiple factors, among which are somewhat decreasing smoking rates and some improvement in diet quality (i.e., decreasing consumption of salt, animal fats and red meat; increasing consumption of fruit, vegetables and fish) [1]. Yet, the impact of these positive changes is likely counterbalanced by an increase in alcohol consumption [1]. In spite of awareness about the role of lifestyle behaviors in chronic disease risk [2], rates of obesity, smoking and alcohol use in Poland are higher than the respective European Union (EU) averages [3]. Currently, overweight (including obesity) concerns 69% of Polish men and 59% of Polish women; moreover, these rates display a growing trend [4]. A report by the Organization for Economic Cooperation and Development (OECD) highlighted that, in Poland, diseases of the circulatory system accounted for a larger share of total mortality compared to the EU average (i.e., 45% versus 36% in 2016), that spending on prevention was relatively low and insufficient physical activity was prevalent [3]. Also, dietary practices are considered a crucial behavior-based factor in premature cardiovascular mortality prevention [5].

Prior to making a dietary choice, especially regarding pre-packaged products, consumers can obtain at-a-glance information about the nutritional value via front-of-package labels (FoPL) whose use has been recommended by the World Health Organization (WHO) [6]. Even though FoPL have been in existence since the late 1980s, research interest in their utility and potential as a public health promotion strategy has been especially salient over the past decade [7], reflecting an effort to combat the increasing rates of overweight/obesity and other non-communicable diseases [8]. The wide variety of FoPL (developed by public or private entities) used around the world can be grouped into two main kinds: those that display numeric data on specific nutrient content (i.e., nutrient-specific labels) and those that synthesize information on ingredient and/or nutrient content into a graphic and/or color-coded logo (i.e., summary labels) [9]. Generally, salient, color-coded and easily comprehensible FoPL have been shown to be effective in steering consumers towards selecting healthier food/beverage options and also in urging manufacturers to engage in product reformulation [6,8,10]. Recently, the European Commission underscored the need to implement a uniform and mandatory FoPL at the EU level [8]. Presently, EU rules stipulate that the provision of nutrition information on FoPL is possible on a voluntary basis [8].

In Poland, there have been voluntary FoPL initiatives involving industry-elaborated models such as Healthy Choice, Guideline Daily Amounts and SENS. For example, the Healthy Choice FoPL was introduced in 2008 as a voluntary and self-regulated action funded and implemented by the food industry [6]. That label accounts for the presence of saturated and trans-fatty acids, sodium and added sugar, distinguishing between basic and discretionary foods [6,11]. The Netherlands was the first country to implement Healthy Choice (in 2006), yet consumers there seemed ambivalent about the color-coding purport and were likewise unsure whether absence of the logo implied product unhealthiness [6]. As a result, in 2016, the Dutch government withdrew its support for that label [6,11] and in 2019 decided to engage in the implementation of NutriScore [12]. The latter has already been adopted in France, Belgium, Spain, Germany, Switzerland and Luxembourg.

In that context, the aim of the present study was to assess Polish consumers’ overall ability to estimate product healthiness through their objective understanding of five FoPL (Health Star Rating (HSR), Multiple Traffic Lights (MTL), NutriScore, Reference Intakes (RI) and Warning Label) in order to provide insights into FoPL models that may be effective in this cultural context. Objective understanding was defined as the individual’s ability to correctly interpret (i.e., as intended) the information provided by the FoPL [13].

## 2. Materials and Methods

### 2.1. Study Design and Participant Recruitment

This analysis is part of an 18-country research project comparing the effectiveness of different FoPL by means of a randomized experiment conducted online [14,15]. The international web panel provider called PureProfile (https://business.pureprofile.com/, accessed on 9 November 2021) was used for the recruitment of approximately 1000 volunteers per country, observing the following quota-based sampling guidelines: 50% females, equal distribution across three age categories (18–30, 31–50 and >51 years) and across three household income levels (low, medium (i.e., within a 33% bracket around the country-specific median) and high), using as reference the World Income Inequality Database [16]. The inclusion criteria and online intervention were identical across all 18 countries.

The main selection criterion for the countries was the presence of a FoPL discourse at the national level. The first wave of recruitment took place in 2018 and included 12 countries (Argentina, Australia, Bulgaria, Canada, Denmark, France, Germany, Mexico, Singapore, Spain, the UK and the US), and the second wave took place in 2019 and included 6 additional countries (Belgium, Italy, the Netherlands, Poland, Portugal and Switzerland) [14,15]. All individuals gave their informed consent for participation before enrollment in the study. Specifically, prior to any data collection, each eligible individual was required to read and agree with a written statement about his/her volunteer participation in the study. Proceeding with the sociodemographic questionnaire implied informed consent. The Institutional Review Board of the French Institute for Health and Medical Research (INSERM) and Curtin University Human Research Ethics Committee both approved the trial protocol. The trial is registered with the Australian New Zealand Clinical Trials Registry (retrospective registration on 20 July 2018; #ACTRN12618001221246; http://www.ANZCTR.org.au/ACTRN12618001221246.aspx, accessed on 9 November 2021).

This study utilized data from the Polish sample (N = 1159), following the CONSORT guidelines.

### 2.2. Intervention

First, we developed mock food packages resembling actual products belonging to three distinct categories: breakfast cereals, cakes and pizza. These food categories have been used as stimuli in other randomized experiments testing FoPL effectiveness in different populations [17,18]. As in other studies, for the present experiment we aimed to select food products that are consumed for various reasons and that represent a range of food types (breakfast meals, ready-to-eat lunch/dinner meals, desserts) and that have sufficient variability so that three nutritionally distinct products for each food category could be created. In order to permit cross-cultural comparisons, we selected food categories that are: (a) consumed in all 18 countries, and (b) frequently advertised on television and online [19,20].

Next, we assigned nutritional quality rating following an examination of food composition databases and real-world products within the nominated product categories. At that step, we identified products of relatively low, intermediate and relatively high nutritional quality on which to base the nutritional profiles of each mock product [21]. For example, in the breakfast cereals category, fat content ranged from 1 g to 16 g, whereas sugar content ranged from 8 g to 30 g per serving; in the pizza category, salt content ranged from 335 mg to 689 mg per serving; in the cakes category, energy (Kcal) ranged from 217 to 463 per serving [21].

Prior to the start of the experiment, the volunteers completed a short pre-trial questionnaire [15]. Then they were asked to view sets of three nutritionally distinct products (all belonging to the fictional brand *Stofer*) within each food category before being asked to perform two ranking tasks. In order to augment the ecological validity of the study, we identified and excluded data from individuals who reported never purchasing ≥ 2 of the three food categories [14]. Likewise, if an individual stated that he or she never bought food items belonging to any of the three food categories, then his/her responses to the corresponding ranking task were left out.

The trial featured two tasks. For the first one, participants viewed sequentially displayed pictures of three sets of three FoPL-free products (i.e., three kinds of breakfast cereal, three kinds of cake, three kinds of pizza). The task was to use one’s objective and/or subjective knowledge and to rank each item according to its nutritional value, as follows: “1 = Highest nutritional quality,” “2 = Medium nutritional quality,” “3 = Lowest nutritional quality.” The pictures did not feature any nutritional value information. Upon completion of the first task, the web panel provider randomized all of the participants to one of five FoPL conditions: RI, Warning Label, HSR, MTL and NutriScore. Then, they were asked to redo the same ranking task. Whereas the same three sets of three food products were viewed, the difference was that this time each picture featured in the lower right-hand corner one of the five FoPL. Our main hypothesis was that the second-ranking task results would be superior to those of the FoPL-free ranking task.

The trial volunteers did not know that they would be viewing the mock products twice or that any FoPL would be presented the second time. The likelihood of any presentation order bias was mitigated by randomizing: (a) the order in which the three food categories appeared on the screen, and (b) the order in which the pictures of the mock products within each category appeared on the screen. Upon completion of the trial, the volunteers responded to a Yes/No/Don’t know question if they remembered having seen a nutrition label.

### 2.3. Description of the FoPL Tested in the Trial

Given the trial objectives, only FoPL models that do not suggest or represent seals of approval (which are also difficult to assess across more than two products at once) were eligible for inclusion in the intervention. Hence, FoPL such as the green “Keyhole” and Healthy Choices were not included. The trial tested the effectiveness of five labels- two summary schemes (HSR and NutriScore) and three nutrient-specific schemes (MTL, RI and Warning Label)—that are currently in use in various countries around the world. The two summary FoPL are based on an algorithm adapted from the British Food Standards Agency Nutrient Profiling System [22]. HSR was introduced in Australia and New Zealand in 2014. It is a monochrome, scaled scheme featuring nutrient-specific information regarding total and saturated fat, sugar, sodium, soluble and/or insoluble dietary fiber, protein and total energy, and ratings going from ½ star (i.e., low nutritional quality) to 5 stars (i.e., high nutritional quality). In turn, the polychromatic NutriScore was introduced in France in 2017 [23]. It takes into consideration total energy, sugar, sodium, saturated fat, dietary fiber (soluble and/or insoluble), protein and the proportion per 100 g or mL of fruit/vegetables/nuts/plant-based oils (walnut, canola, olive) and displays five levels of overall nutritional value, indicated by color–letter combinations ranging from dark green paired with the letter A (i.e., high nutritional quality) to red paired with the letter E (i.e., low nutritional quality) [24].

Next, the RI model was introduced in 2014 by Europe’s food and drink industry as a replacement of the Guideline Daily Amounts model. RI is a monochromatic scheme that uses as reference the recommended intake for an average adult; it provides numeric information regarding the quantity/proportion of total energy, total and saturated fat, sugars and sodium, per portion and per 100 g or mL [25]. In turn, the MTL model is also a nutrient-specific FoPL that the UK government endorsed in 2013; it combines RI and color-coding of the portion-specific quantity of total energy, total and saturated fat, sugars and sodium. Nutrient-specific quantities according to established thresholds (per 100 g or mL) are first calculated and then represented by the colors green (low amount), amber (average amount) or red (high amount) [26,27]. The final FoPL used in the trial was the Warning Label. It was introduced on a mandatory basis in Chile in 2016; it is now part of that country’s Food Labeling and Marketing Law [28]. The Warning Label is a monochromatic scheme that flags food containing either a large amount of energy or an increased amount of one of the nutrients implicated in chronic disease risk (e.g., sugar, salt, saturated fat) [28].

**Figure 1:**
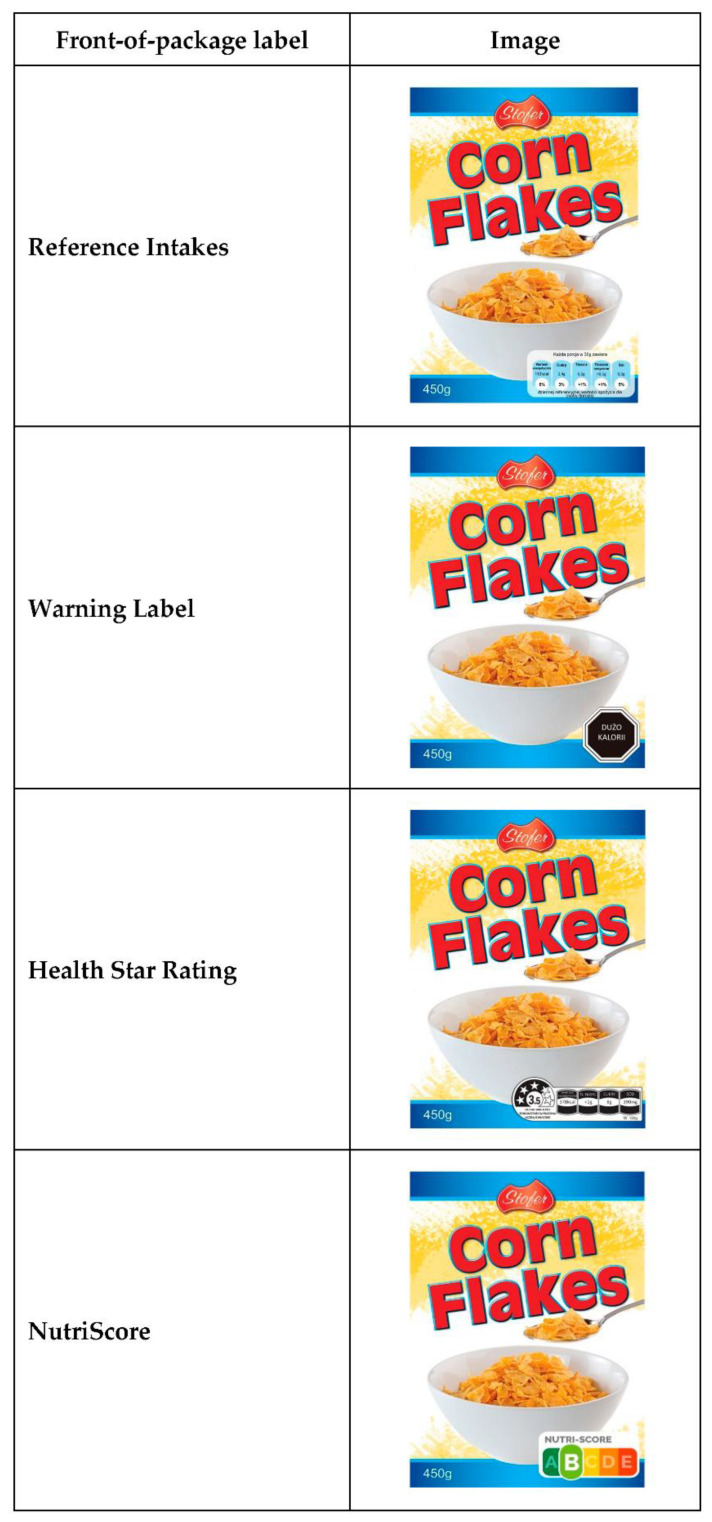

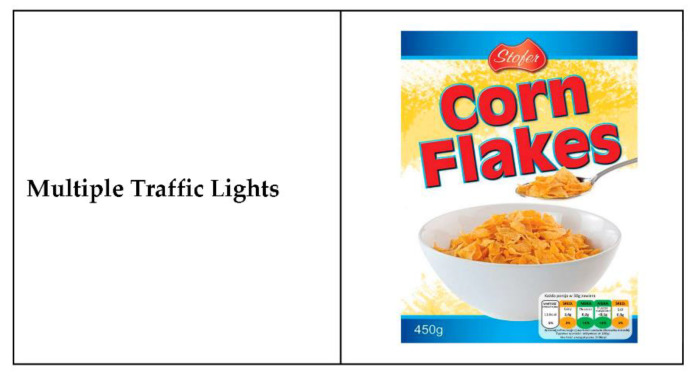
One type of breakfast cereal showing the five front-of-package labels tested in the trial.

The five different FoPL, as featured on one of the mock products (breakfast cereal), is shown in Figure 1. Supplemental Table S1 details the information available on each FoPL. Each label appeared in the same position and covered about the same surface area on the package.

### 2.4. Statistical Analysis

The pre-trial questionnaire [15] provided the following self-reported information used for sample description and statistical adjustment: sex, age, household income (low, medium, high), education (up to high school, trade certification or equivalent, undergraduate or graduate degree), presence of children aged <14 years in the household, grocery shopping responsibility (yes, no, shared), knowledge about nutrition (none, very limited, average, extensive) and perceived quality of own diet (very unhealthy, mostly unhealthy, mostly healthy, very healthy). The sample’s descriptive characteristics are summarized as percentages or as mean values (SD) obtained from Chi-squared tests and ANOVA, respectively. In the main analysis, we compared the results of the two ranking tasks; that was the measure of objective understanding of FoPL (principal outcome). For each participant, we computed the change (expressed in percent and used as an intermediate value) in the number of correct responses within each food category and also across the three food categories. If all three mock products were ranked in the expected order according to their nutritional quality, then ranking was considered correct. Raw scores per food category were as follows: −1 = deterioration, 0 = no change, +1 = improvement. For each task (i.e., without and with FoPL), we added up the raw scores for all food categories; that led to a final score in the range of −3 to +3 points. This was the main outcome variable in the analysis. For each food category and also for all three food categories combined, we estimated the association between FoPL exposure and change in product ranking ability via multivariable ordinal logistic regression. In these models, we used RI as reference. Odds ratios (OR) and 95% confidence intervals (CI) are reported.

Following analysis of the full sample, we tested for interaction by sex, age, education and income group, respectively. In order to account for any bias introduced by multiple comparisons in the subgroup analyses, we corrected the significance level via the false discovery rate method. Finally, in order to evaluate the robustness of the main results, we carried out a sensitivity analysis using only data from volunteers who remembered seeing a FoPL during the experiment.

All statistical tests were performed using SAS 9.4 (SAS Institute Inc., Cary, NC, USA), applying a 0.05 significance level in the full sample analysis and 0.01 in the age-stratified analyses.

## 3. Results

### 3.1. Sample Description

The sample comprised 1159 Polish adults (mean age = 40.9 ± 15.4 years). Exclusive or shared grocery shopping responsibility was reported by 97.0% of the sample, whereas 77.9% self-evaluated their diet as mostly or very healthy. Participant characteristics in the full sample and by age category (18–30, 31–50, 51–89 years) are presented in Table 1.

### 3.2. Effect of FoPL Condition on Product Rankings

Table 2 presents the multivariable ordinal logistic regression results derived from the full sample.

As regards breakfast cereals, and compared with RI, significant changes in objective understanding of FoPL as evidenced by product ranking ability were seen for NutriScore (OR = 1.89; 95% CI: 1.18–3.02; *p* < 0.008) and HSR (OR = 1.62; 95% CI: 1.01–2.59; *p* < 0.05). In the cakes category, significant changes in product ranking ability, compared with RI, were found for three FoPL: NutriScore (OR = 2.85; 95% CI: 1.79–4.53; *p* < 0.0001), Warning Label (OR = 2.39; 95% CI: 1.49–3.81; *p* < 0.0003) and HSR (OR = 1.83; 95% CI: 1.14–2.93; *p* < 0.01). In the pizza category, significant change in product ranking ability, compared with RI, was seen only for the group randomized to NutriScore (OR = 1.59; 95% CI: 1.02–2.46; *p* < 0.04).

When modeling all three food categories together, significant changes in ranking ability compared with RI, were seen for NutriScore (OR = 2.02; 95% CI: 1.41–2.91; *p* < 0.0001) and Warning Label (OR = 1.61; 95% CI: 1.12–2.32; *p* < 0.01). Thus, NutriScore emerged as the only FoPL that was able to elicit favorable changes in ranking ability both across and within food categories, relative to RI. In contrast, no significant associations within or across food categories were observed for MTL.

The only statistically significant interaction was found between FoPL exposure and age (*p* < 0.05), hence we proceeded with age-stratified analyses (Table 3).

Significant associations were observed mainly in the youngest age group (aged 18–30 years). Compared to RI, a favorable change in ranking ability when modeling all three food categories together was seen among young adults randomized to NutriScore (OR = 3.88; 95% CI: 2.04–7.36; *p* < 0.0001). Lesser yet significant improvement was also seen among adults aged ≤ 50 years randomized to Warning Label. When modeling each food category separately, significant associations again emerged mainly among young adults and only in the cakes category. Specifically, compared to RI, the largest improvement in ranking ability was found among young adults randomized to NutriScore (OR = 6.88; 95% CI: 3.05–15.51; *p* < 0.0001); that was followed by young adults randomized to Warning Label (OR = 5.07; 95% CI: 2.20–11.66; *p* < 0.0001). Some improvement in ranking ability was also found among adults aged 31–50 years randomized to Warning Label (OR = 2.52; 95% CI: 1.09–5.83; *p* < 0.05).

In order to assess the robustness of these findings, we first excluded data from 336 individuals who did not remember having seen a FoPL during the second task and then refit the models. This sensitivity analysis largely produced the same results (data not tabulated).

## 4. Discussion

Using a quota-based sample of the Polish population, this online trial revealed that—relative to RI—NutriScore was the only FoPL able to elicit some favorable change in product ranking ability among consumers, both across and within food categories. Specifically, across the three food categories, significant improvement in objective understanding of FoPL, measured by one’s ability to rank the products according to their nutritional value, was found in the group randomized to NutriScore and—to a lesser extent—in the group randomized to Warning Label. Next, NutriScore was the only FoPL associated with favorable change in product ranking ability as regards the pizza products. For breakfast cereals and cakes, significant changes in product ranking ability compared with RI were respectively seen for NutriScore and HSR, and for NutriScore, Warning Label and HSR.

The findings of this randomized experiment also supported variability in the objective understanding of FoPL by age group. In age-stratified analyses, significant associations were the most likely to be observed in the youngest age group (aged 18–30 years), where NutriScore emerged as the only label capable of improving the objective understanding of FoPL relative to RI. When modeling each food category separately, significant improvements in objective understanding were observed with NutriScore and with Warning Label as regards assessment of the nutritional value of cakes. No significant associations emerged as regards the other two food products or in the oldest age group. The latter might be partly due to digital literacy considerations and/or familiarity with the pre-packaged food products used in the trial. Objective understanding of FoPL either within or across food categories did not seem to improve with the addition of MTL, irrespective of age.

The present analysis is part of an international research project on FoPL effectiveness among adult consumers recruited from the general population [14,15]. Across all 18 countries and across all three food categories, NutriScore performed best, even in countries (Australia, the UK) where alternative official labels have been in use for some time (e.g., HSR, MTL) [14]. Yet, when comparing the present findings with those obtained in the other Eastern European country (Bulgaria) included in the experiment, some differences emerge. For example, no significant associations with Warning Label were found either within or across food categories among Bulgarian consumers [29], whereas among their Polish counterparts, that FoPL showed significant improvement in objective understanding across food categories and within the cakes category, relative to RI. In general, awareness about healthy eating, familiarity with FoPL and the scope/duration of the public discourse about food labeling might help explain country-specific FoPL effects [13,30]. Polish consumers have been familiar with RI and, since 2008, with the Healthy Choice label [6]. Another characteristic of Polish consumers identified in previous research pertains to a tendency to rate various food products as healthier compared to the respective ratings by consumers in other countries [31].

Overall, consumers seem to prefer simple FoPL requiring a relatively low cognitive load and enabling quick processing given that point-of-purchase decisions are made rapidly [32]. Thus, the polychromatic design and especially the green-to-red scale—which has been highlighted as a promising element of nutrition policy strategies [33]—might partly explain the superior performance of NutriScore relative to other FoPL [34]. A study with 1500 Polish adults carried out in 2017–2018 and comparing different FoPL revealed that the MTL received the highest rating, while the green “Keyhole” received the lowest rating by consumers; in turn, NutriScore, with its simplicity and clarity, appealed to the majority of consumers, especially to those with less formal education [35]. The effect on food purchases of NutriScore, along with three other labels, was recently tested in a 10-week real-life grocery shopping experiment in 60 supermarkets in France [36]. The authors noted that NutriScore emerged as the most effective FoPL, highlighting its ability to attract attention and help shoppers rank food/beverage products by nutritional quality [36]. Eye-tracking research has linked color-coding with quicker detection and attention to FoPL, especially among individuals without explicit nutritional goals [37]. Further, a study testing various FoPL on sweet and savory snacks in a sample of 1000 German and Polish volunteers found that color-coding was an efficient strategy for augmenting one’s self-perception of being able to select healthful options [30].

Another advantageous feature of FoPL might be the choice of the reference amount (i.e., per portion, per 100 g, etc.). For example, research in 16 European countries documented that information about total energy was best understood when it was provided per 100 g [38]. In the same study, 23.5%, 3.8% and 3.5% of the Polish participants were able to correctly interpret the reference amount when it was presented per 100 g, per 100 Kcal and per portion, respectively [38]. Another international study provided evidence that product healthfulness evaluations remained virtually unchanged following the addition of percentages of proposed daily reference quantities to the label [31].

Regarding the study context, several recent publications have indicated that, in general, the overall nutritional quality of the Polish diet could not be considered as high despite promising evidence of improvement in diet quality in some population strata [1]. For example, a 2019 OECD report noted that 42% of Polish adults did not consume fruit or a portion of vegetables on a daily basis [3]. Next, results of the National Multicenter Health Survey (WOBASZ II, 2013–2014) involving a random sample of 5690 Polish adults aged 20 years and older revealed that slightly more than half of the participants believed that their diet was appropriate, while in fact 60% had a low-quality diet, 15% had a healthy diet, 8% had a low-fat/low-cholesterol diet and 1% had a low-calorie diet [39]. In the present analysis, close to 80% of the sample perceived their diet as being mostly or very healthy. The WOBASZ II study also revealed a high prevalence of a number of diet-related chronic conditions such as obesity, hypertension, diabetes and hypercholesterolemia [39]. Interestingly, some qualitative and quantitative studies showed that overweight and obese individuals were more likely to report a need for a FoPL compared to their normal-weight counterparts [40,41], whereas a recent study conducted among 1051 Polish consumers showed that sociodemographic and anthropometric characteristics were not significant predictors of reading food labels [42]. Another Polish study with 1017 adults revealed that those with food neophobia were more likely to report not consulting food labels compared to their counterparts without food neophobia [43]. It should also be noted that prior research did not identify the cultural context as a strong predictor of the general perception of FoPL [44].

The absence of an actual grocery shopping experience is seen as a limitation of this study, as is its reliance on a quota-based sample. However, in the context of an online experiment, the role of factors that can impact food choices (i.e., shopping habits, cost, time pressure, familiarity, brand loyalty, availability of ingredients lists, etc.) was likely diminished. In the trial, we used a fictional brand called *Stofer* and did not provide any nutrition-related guidance (e.g., organic certification, country of production). Next, the ranking tasks might have entailed some bias. However, we largely followed the conceptual framework outlined by Grunert et al. [13] with its focus on the construct of objective understanding of FoPL. Despite some variability in the specific objectives of different FoPL schemes, they all represent population-level strategies to improve people’s diets, seeking to inform consumers of the relative healthiness of food products and facilitate improved product choices; consumer understanding of different FoPL has been investigated in several randomized trials [17,45]. Also, prior quantitative and qualitative research has shown evidence for gradation in FoPL models, such as Warning Label [45,46,47]. Finally, the lack of data on health status, personal dietary needs and/or motivations of the volunteers is also seen as a limitation of the study.

The use of three sets of three products, thus reducing the possibility of correct responses by chance while approximating real-life shopping situations, is seen as a salient strength of the trial. The food categories (breakfast cereals, cakes and pizza) were chosen as stimuli because they are available, familiar, relatively affordable, consumed in all 18 countries, and present substantial nutritional value variability [14,21].

## 5. Conclusions

FoPL are increasingly being recognized as useful supplementary strategies in the fight against diet-related chronic diseases, and the European Commission intends to adopt a uniform FoPL model by the end of 2022 [8,48]. Supporting the study hypothesis, the trial results provided some evidence that the nutrition information conveyed by certain FoPL could improve Polish consumers’ ability to correctly rank food products according to nutritional quality. Compared to RI, NutriScore—which is a simple polychromatic summary FoPL—emerged as the only FoPL that was able to produce some favorable change in product ranking ability among Polish consumers, both across and within food categories. These findings are of importance for the ongoing debate about the need for and choice of an EU-wide uniform and mandatory FoPL model.

## Figures and Tables

**Table 1:** Descriptive characteristics of the full sample and by age group.

	Full Sample (N = 1159)		Ages 18–30 years (N = 390)		Ages 31–50 years (N = 389)		Ages 51–89 years (N = 380)		*p*
Randomization group									0.97
Health Star Rating	232	(20.0)	77	(19.7)	79	(20.3)	76	(20.0)	
Multiple Traffic Lights	231	(19.9)	79	(20.3)	71	(18.2)	81	(21.3)	
NutriScore	232	(20.0)	80	(20.5)	83	(21.3)	69	(18.2)	
Reference Intakes	232	(20.0)	76	(19.5)	78	(20.1)	78	(20.5)	
Warning Label	232	(20.0)	78	(20.0)	78	(20.1)	76	(20.0)	
Mean age, years (SD)	40.9	(15.4)	24.9	(3.5)	38.3	(5.4)	60.1	(6.6)	
Sex									<0.0001
Male	579	(50.0)	123	(31.5)	206	(53.0)	250	(65.8)	
Female	580	(50.0)	267	(68.5)	183	(47.0)	130	(34.2)	
Education									<0.0001
Up to high school	494	(42.6)	161	(41.3)	148	(38.1)	185	(48.7)	
Trade certification	122	(10.5)	16	(4.1)	55	(14.1)	51	(13.4)	
Undergraduate level	192	(16.6)	105	(26.9)	58	(14.9)	29	(7.6)	
Graduate level	351	(30.3)	108	(27.7)	128	(32.9)	115	(30.3)	
Household income									<0.0001
Low	375	(32.4)	103	(26.4)	170	(43.7)	102	(26.8)	
Medium	397	(34.2)	185	(47.4)	127	(32.7)	85	(22.4)	
High	387	(33.4)	102	(26.2)	92	(23.6)	193	(50.8)	
Children ≤ 14 years in household									<0.0001
No	648	(55.9)	185	(47.4)	148	(38.1)	315	(82.9)	
Yes	511	(44.1)	205	(52.6)	241	(61.9)	65	(17.1)	
Grocery shopping responsibility									0.005
No	35	(3.0)	11	(2.8)	16	(4.1)	8	(2.1)	
Shared	291	(25.1)	84	(21.5)	87	(22.4)	120	(31.6)	
Yes	833	(71.9)	295	(75.7)	286	(73.5)	252	(66.3)	
Knowledge about nutrition									0.02
Very limited	168	(14.5)	48	(12.3)	68	(17.5)	52	(13.7)	
Average level	852	(73.5)	284	(72.8)	289	(74.3)	279	(73.4)	
High level	139	(12.0)	58	(14.9)	32	(8.2)	49	(12.9)	
Self-assessed diet quality									0.68
Mostly or very unhealthy	256	(22.1)	81	(20.8)	91	(23.4)	84	(22.1)	
Mostly or very healthy	903	(77.9)	309	(79.2)	298	(76.6)	296	(77.9)	

Values refer to number (% in parentheses) except when noted otherwise. *p*-values obtained from Chi-squared tests or ANOVA, as appropriate.

**Table 2:** Assessment of objective understanding of FoPL as measured by nutritional quality ranking before (without FoPL) and after randomization (with FoPL); N = 1159.

Food Category	Health Star Rating			Multiple Traffic Lights			NutriScore			Warning Label		
	OR	(95% CI)	*p*	OR	(95% CI)	*p*	OR	(95% CI)	*p*	OR	(95% CI)	*p*
All categories	1.25	(0.87–1.81)	0.23	0.96	(0.66–1.39)	0.83	2.02	(1.41–2.91)	0.0001	1.61	(1.12–2.32)	0.01
Cereals	1.62	(1.01–2.59)	0.05	1.26	(0.78–2.02)	0.34	1.89	(1.18–3.02)	0.008	1.51	(0.94–2.44)	0.09
Cakes	1.83	(1.14–2.93)	0.01	1.38	(0.86–2.23)	0.19	2.85	(1.79–4.53)	<0.0001	2.39	(1.49–3.81)	0.0003
Pizzas	0.89	(0.56–1.39)	0.60	0.68	(0.43–1.07)	0.09	1.59	(1.02–2.46)	0.04	1.22	(0.78–1.91)	0.37

Multivariable ordinal logistic regression (“Reference Intakes” = reference) with adjustment for sex, age, education, household income, children < 14 years in household, grocery shopping responsibility, self-assessed diet quality and knowledge about nutrition. CI, confidence interval; FoPL, front-of-package label; OR, odds ratio.

**Table 3:** Age-specific objective understanding of FoPL as measured by nutritional quality ranking before (without FoPL) and after randomization (with FoPL).

Food Category	Health Star Rating		Multiple Traffic Lights		NutriScore		Warning Label	
	OR	(95% CI)	OR	(95% CI)	OR	(95% CI)	OR	(95% CI)
**Ages 18–30 years**								
All categories	1.51	(0.79–2.90)	0.94	(0.49–1.80)	3.88	(2.04–7.36)	2.12	(1.11–4.06)
Breakfast cereals	2.91	(1.27–6.68)	1.34	(0.58–3.14)	3.21	(1.40–7.33)	2.11	(0.90–4.96)
Cakes	2.29	(0.99–5.28)	1.26	(0.54–2.93)	6.88	(3.05–15.51)	5.07	(2.20–11.66)
Pizzas	0.97	(0.44–2.14)	0.70	(0.32–1.55)	1.92	(0.90–4.08)	0.90	(0.40–1.99)
**Ages 31–50 years**								
All categories	1.07	(0.57–2.01)	0.95	(0.49–1.82)	1.60	(0.86–2.98)	1.99	(1.06–3.73)
Breakfast cereals	1.23	(0.55–2.76)	2.29	(0.57–2.93)	1.48	(0.67–3.28)	1.94	(0.88–4.30)
Cakes	1.74	(0.75–4.05)	1.65	(0.69–3.97)	2.00	(0.87–4.60)	2.52	(1.09–5.83)
Pizzas	0.75	(0.33–1.68)	0.58	(0.25–1.32)	2.02	(0.95–4.34)	1.62	(0.74–3.56)
**Ages 51–89 years**								
All categories	1.18	(0.61–2.25)	1.03	(0.54–1.94)	1.31	(0.68–2.53)	1.05	(0.55–2.02)
Breakfast cereals	1.18	(0.50–2.78)	1.31	(0.57–3.04)	1.57	(0.66–3.76)	0.85	(0.35–2.09)
Cakes	1.75	(0.76–4.01)	1.31	(0.58–2.30)	1.66	(0.72–3.85)	1.38	(0.59–3.20)
Pizzas	0.77	(0.35–1.70)	0.76	(0.35–1.67)	0.90	(0.41–1.97)	1.06	(0.48–2.32)

Multivariable ordinal logistic regression (“Reference Intakes” = reference) with adjustment for sex, age, education, household income, children < 14 years in household, grocery shopping responsibility, self-assessed diet quality and knowledge about nutrition. CI, confidence interval; FoPL, front-of-package label; OR, odds ratio.

## Data Availability

The data supporting the results of this study are included in the present article.

## Supplementary Materials

The following supporting information can be downloaded at: https://www.mdpi.com/article/10.3390/foods11010134/s1, Supplemental Table S1: Polish to English translation of the information featured on each FoPL as displayed on one type of breakfast cereal.
