# Supplementary material for: Polish Consumers’ Understanding of Different Front-of-Package Food Labels: A Randomized Experiment
Source: Foods. 2022 Jan 5;11(1):134. doi: 10.3390/foods11010134 (PMC8750026; doi:10.3390/foods11010134)
Supplement: Supplementary file 1 [file foods-11-00134-s001.zip › foods-1480890-supplementary.pdf]

Table S1. Polish to English translation of the information featured on each FoPL as displayed on one type of breakfast cereal.

Reference Intakes	Warning Label	Health Star Rating	Nutri-Score	Multiple Traffic Lights
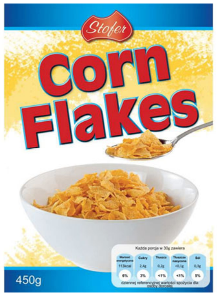	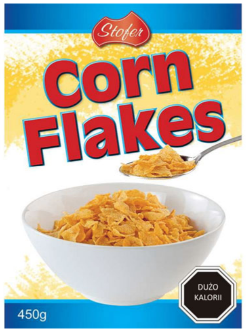	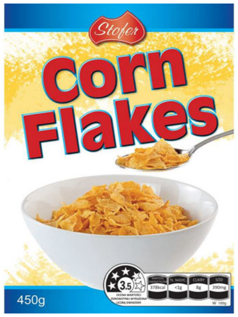	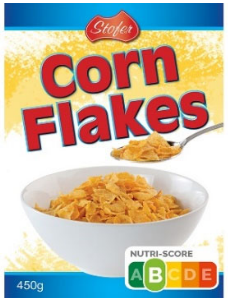	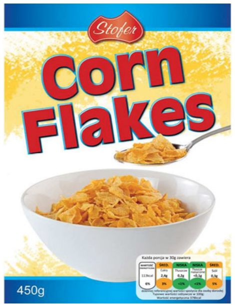
Each portion of 30g contains:<br><br>Energy value: 113kcal (6%)<br>Sugars: 2,4g (3%)<br>Fat: 0,2g (<1%)<br>Saturated fat: <0,1g (<1%)<br>Salt: 0,3g (5%)<br><br>*Daily reference intake for an adult*	High in calories	Health value rating expressed as number of stars: 3,5<br><br>Energy value: 378kcal<br>Saturated fat: <0,1g<br>Sugars: 8g<br>Sodium: 390mg<br><br>per 100g	B	Each portion of 30g contains:<br><br>Energy value: 113kcal (6%)<br>Sugars: 2,4g (3% - medium)<br>Fat: 0,2g (<1% - low)<br>Saturated fat: <0,1g (<1% - low)<br>Salt: 0,3g (5% - medium)<br><br>*Daily reference intake for an adult*<br>*Typical nutritional value per 100g: 378kcal*
